# Access to reconstructive plastic surgery for patients undergoing bariatric surgery in the Unified Health System (SUS)

**DOI:** 10.1590/0100-6991e-20233520-en

**Published:** 2023-07-19

**Authors:** MURILO SGARBI SECANHO, WILSON CINTRA, IGOR CASTRO CARNEIRO, GUILHERME FREDERICO FERRO ALVES, ROLF GEMPERLI

**Affiliations:** 1 - Hospital das Clínicas da Universidade de São Paulo (USP), Disciplina de Cirurgia Plástica - São Paulo - SP - Brasil

**Keywords:** Surgery, Plastic, Brazil, Bariatric Surgery, Public Health, Cirurgia Plástica, Brasil, Cirurgia Bariátrica, Saúde Pública

## Abstract

**Introduction:**

obesity is one of the most common diseases worldwide, and the most effective treatment to it is the bariatric surgery. One of the negative impacts of this procedure is the body dysmorphia caused by overhanging skin. In Brazil, the national health system - Sistema Único de Sáude (SUS) - provide body contouring surgery to treat post-bariatric patients, since 2007. This article aims to describe the Brazilian public health approach to post bariatric patients and perform an analyze in the Brazilian health care database.

**Methods:**

in Brazilian Health System database, a search for the post-bariatric procedures performed between 2007 to 2021 was done. The variables analyzed were geographic location, year, mean days of hospitalization, death, and mortality rate. Also, we evaluated the number of bariatric procedures done in the same period. Statistical analysis was performed using the Student-t and the chi-square tests and p-value <0.5 was considered significant.

**Results:**

a total of 12,717 plastic surgery procedures in post bariatric patients were done, with a national prevalence of 13.8%. Dermolipectomy was the most performed procedure, with 6,719. The years of 2020 and 2021 suffered a decreased of 64.3% and 70.9% in the number of surgeries (p<0,001). Bariatric Procedures had a high rate and a higher percentage of growth comparing to post bariatric surgery (p<0,001), totalizing 93,589 surgeries.

**Conclusions:**

Brazil had a significant number of body contouring surgery, however with a low prevalence. Dermoliepctomy was the most common procedure performed. We could notice a significant impact of COVID pandemic in those procedures .

## RESUMO

**Introdução:**

obesidade é uma das doenças mais comuns do mundo e a cirurgia bariátrica é o tratamento mais eficaz até o momento. Um dos impactos negativos do procedimento é o dismorfismo corporal causado pelo excesso cutâneo. No Brasil, o Sistema Único de Saúde (SUS) promove cirurgia de contorno corporal para tratar pacientes com perda ponderal maciça desde 2007. Este artigo tem como objetivo descrever a abordagem do SUS para pacientes pós-bariátricos através de análise do banco de dados do próprio sistema.

**Métodos:**

foi realizada pesquisa no banco de dados do SUS para procedimentos em pós-bariátricos entre 2007 e 2021. As variáveis analisadas foram localização geográfica, ano, tempo médio de internação, morte e taxa de mortalidade. Também analisamos o número de procedimentos bariátricos realizados no mesmo período. Análise estatística foi realizada utilizando os testes t de Student e do Qui-Quadrado, considerando valor p<0,5 como significante.

**Resultados:**

um total de 12.717 procedimentos de cirurgia plástica foram realizados em pacientes pós-bariátricos, com prevalência nacional de 13,8%. Dermolipectomia foi o procedimento mais realizado, com um total de 6.719. Nos anos de 2020 e 2021 houve um decréscimo de 64,3 e 70,9% no total de cirurgias, respectivamente (p<0,001). Procedimentos bariátricos tiveram maior número total e taxa de crescimento maiores do que cirurgia pós-bariátricas (p<0,001), totalizando 93.589 cirurgias.

**Conclusões:**

houve um número significativo de cirurgias de contorno corporal no Brasil, embora com baixa prevalência. Dermolipectomia foi o procedimento mais realizado. Notamos impacto significativo da pandemia Sars-CoV-2 no total de procedimentos realizados.

## INTRODUCTION

The most effective treatment for obesity is bariatric surgery. Despite all its benefits, it has one negative impact, body dysmorphia, caused by residual skin flaccidity after weight loss. This change can lead to aesthetic, functional, and psychological damage. Fungal infections, ulcers, and poor hygiene are frequent problems. Depression, anxiety, negative body image, low self-esteem, and worsened quality of life affect mental health. All these problems, isolated or combined, can lead to weight regain in these patients^1^ ^,^ ^2^.

Body contouring surgery is the only effective treatment for the excess skin left by massive weight loss. Considered a functional and aesthetic procedure, it combines a range of surgeries, such as Mastopexy, Abdominoplasty, Brachioplasty, and Cruroplasty^3^. The incidence of these procedures has been increasing, making them safer and with a lower rate of complications^4^. These surgeries positively impact the quality of life and body image of such patients, reducing depression and improving weight maintenance. For all these considerations, body contouring surgery plays a fundamental role in the multidisciplinary approach of patients with weight loss^5^ ^,^ ^6^.

In Brazil, the Unified Health System (SUS) has been offering this treatment since 2007. Founded in 1988, SUS provides free medical care in public institutions. One of its fundamental concepts is integrity, which means that all treatments must be offered in a complete way, with the entire multidisciplinary approach^7^. Based on this concept, reconstructive plastic surgery acts as one of the pillars of the SUS approach to patients undergoing surgery to treat obesity.

Despite the availability of these procedures, the Brazilian health system is not free of limitations. As a continental and middle-income country, Brazil suffers from regional disparities in its medical structure and workforce, leading to a difference in the provision of health services. Another important point is that international crises affect the Brazilian economic system, indirectly impacting SUS’s capacity. During the last two years, the impact of the Sars-Cov-2 pandemic led to a financial and health system crisis in Brazil, resulting in delays and suspensions of several elective surgeries, including bariatric and reconstructive procedures^9^ ^-^ ^11^.

Several reports in the literature have already described national health system approaches for patients with severe weight loss after bariatric surgery, mainly in North America and Europe^12^ ^-^ ^17^.

SUS is known worldwide as one of the largest public health systems. We believe that the Brazilian experience can bring new perspectives and questions on the subject. This article aims to evaluate the approach of the Brazilian Public Health System to post-bariatric patients and analyze the national database.

## METHODS

We conducted the research on the DATASUS website, the SUS database, using the codes referring to each procedure, in the period between 2007 and 2021. The variables analyzed were geographic location (region and state), year, average length of stay, and mortality rate. We also analyzed the number of bariatric procedures performed in the same period.

All variables were included in a Microsoft Excel^©^ spreadsheet. Statistical analysis was complemented by the Student’s t and chi-square tests, considering a p-value significant when <0.5.

[Table t1]

**Table 1 t1:** Inclusion criteria for post-bariatric surgery in the Unified Health System.

Inclusion criteria	
	Adherence to follow-up after bariatric surgery
Mammaplasty	Functional restrictions due to breast ptosis, with spinal imbalance.
	Recurrent skin infections in the mammary folds, fungal or bacterial.
	Psychopathological changes due to weight changes (psychiatric criterion).
Dermolipectomy	Functional restrictions by the apron-like abdomen fold and spinal imbalance.
	Recurrent skin infections, fungal or bacterial
	Psychopathological changes due to weight changes (psychiatric criterion).
Brachioplasty and Cruroplasty	Professional limitation due to weight changes and skin excess.
	Recurrent skin infections, fungal or bacterial
	Psychopathological changes due to weight changes (psychiatric criterion).

## RESULTS

A total of 12,717 post-bariatric plastic surgeries were performed between 2007 and 2021, with a national prevalence of 13.8%. Dermolipectomy was the most performed procedure, with 6,719 cases (Table 2).

**Table 2:** Number of post-bariatric procedures performed in Brazil since 2007.

Procedure	No.
Dermolipectomy	6,719
Brachioplasty	1,513
Cruroplasty	1,494
Mammaplasty	2,491
Circumferential Dermolipectomy	500
Total	12,717

[Table t3]

**Table 3 t3:** Number of reconstructive plastic surgeries in patients with major weight loss after bariatric surgery, by region.

Region	n (%)
North	210 (1.6)
Northeast	1,418 (11.1)
Midwest	849 (6.7)
Southeast	6,316 (49.7)
South	3,924 (30.9)
Total	12,717

The mean length of hospital stay was 1.7 days, with an increase to 2.1 when considered dermolipectomy alone. There were three deaths, two in patients undergoing dermolipectomy and one in reduction mammoplasty.

The Southeast region had 49.7% of all procedures. The states with most of procedures were São Paulo, with 4,159 surgeries, and Santa Catarina, with 1,443. Amapá, Rondônia, and Roraima, three Northern states, did not perform any procedure. Among the states that offered the treatment, 66.7% of the surgeries took place in the capitals.

**Figure 1:**
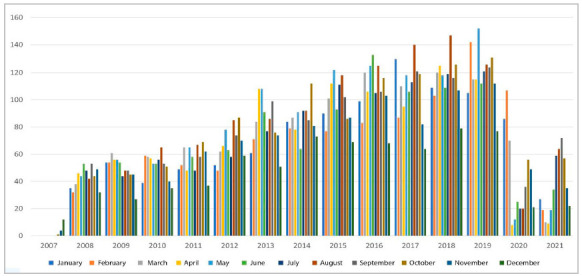
Postbariatric procedures in Brazil, by year and month, since 2007.

The year 2019 had the highest rate of procedures, and 2020 and 2021, the lowest. The difference between years was statistically significant (p<0.001) (Figure 1). Compared with 2019, the last year before the Sars-Cov-2 pandemic, there were decreases of 64.3% and 70.9% in the years 2020 and 2021, respectively.

**Figure 2:**
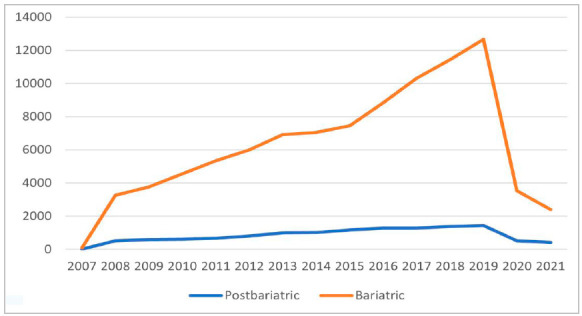
Postbariatric procedures versus bariatric surgeries performed in Brazil, since 2007.

There was a greater number of bariatric procedures, and the growth rate of this type of surgery in relation to the reconstructive one was higher (p<0.001) (Figure 2), totaling 93,589 surgeries. Compared with body contouring surgery, there was a 1.47 fold increase in bariatric surgeries in Brazil. The two types of procedures were more prevalent in the South and Southeast, and most were performed in state capitals.

## DISCUSSION

Since the approval of body contouring procedures for patients after massive weight loss by the Brazilian Ministry of Health in 2008, there has been a progressive increase in the number of plastic surgeries in post-bariatric patients, with a decrease in numbers during the Sars-Cov-2 pandemic. However, Brazil has a significant difference between the numbers of bariatric and post bariatric surgeries performed.

Among all surgeries offered by SUS, dermolipectomy had the highest prevalence, in agreement with other publications^4^ ^,^ ^13^ ^,^ ^18^. Excess skin on the abdomen is the most frequent complaint of patients, and this leads to discomfort, physical limitations, and dermatological problems. Several studies show the positive impact of these procedures on the quality of life, personal hygiene, and ambulation of such patients^6^.

Analogously to other countries - Poland, Spain, Austria, and Italy -, Brazil offers free body contouring procedures through SUS^14^ ^,^ ^16^ ^,^ ^19^ ^,^ ^20^. Inclusion criteria are similar to those proposed by the American Society of Plastic Surgery (ASAPS), mainly related to comorbidities and skin redundancy, unlike countries such as Italy, Norway, and England, which use BMI as an inclusion criterion^3^ ^,^ ^20^ ^,^ ^21^.

[Table t4]

**Table 4 t4:** Type of reconstructive plastic surgery in patients with major weight loss after bariatric surgery, by state.

Procedure	Dermolipectomy	Brachioplasty	Cruroplasty	Mammaplasty	Circumferential Dermolipectomy	Total
Rondônia	1	0	1	0	0	2
Acre	16	1	0	50	5	72
Amazônia	0	1	0	0	0	1
Pará	78	11	10	20	0	119
Tocantins	13	1	1	-	1	16
Maranhão	46	14	14	18	4	96
Ceará	61	17	14	27	4	123
Rio Grande do Norte	18	10	3	1	0	32
Paraíba	29	8	8	4	0	49
Pernambuco	365	153	122	238	13	891
Alagoas	21	4	5	7	0	37
Sergipe	16	5	4	4	0	29
Bahia	76	27	28	29	1	161
Minas Gerais	617	180	184	247	27	1,255
Espírito Santo	299	54	27	61	50	491
Rio de Janeiro	230	57	55	60	12	414
São Paulo	2,146	538	519	872	81	4,156
Paraná	1,139	108	129	161	211	1,748
Procedure	Dermolipectomy	Brachioplasty	Cruroplasty	Mammaplasty	Circumferential Dermolipectomy	Total
Santa Catarina	748	106	183	362	44	1,443
Rio Grande do Sul	302	103	102	184	42	733
Mato Grosso do Sul	205	52	54	48	3	362
Mato Grosso	154	33	8	65	1	261
Goiás	84	12	9	19	0	124
Distrito Federal	55	18	14	14	1	102
Total	6,719	1,513	1,494	2,491	500	12,717

[Table t5]

**Table 5 t5:** Type of reconstructive plastic surgery in patients with major weight loss after bariatric surgery, by year.

Year	Dermolipectomy	Brachioplasty	Cruroplasty	Mammaplasty	Circumferential Dermolipectomy	Total
2007	15	0	1	1	0	17
2008	342	41	46	87	0	516
2009	341	84	67	100	0	592
2010	313	104	96	106	0	619
2011	396	95	104	83	0	678
2012	450	108	106	138	0	802
2013	555	137	106	176	12	986
2014	559	131	118	182	28	1,018
2015	598	160	142	190	78	1,168
2016	645	130	143	241	130	1,289
2017	602	142	166	317	58	1,285
2018	709	140	146	303	80	1,378
2019	736	141	162	321	72	1,432
2020	251	47	53	135	24	510
2021	207	53	38	111	18	427
Total	6,719	1,513	1,494	2,491	500	12,717

The use or not of the body mass index is controversial, since high BMIs are associated with greater surgical complications^22^. Skin flaccidity leads to symptoms and complaints that limit daily activities, such as skin lesions due to friction and difficulty in walking, so these patients must undergo procedures to improve these complaints and quality of life, regardless of BMI, if they have control of comorbidities and adequate surgical preparation^24^.

The minimum age to undergo plastic surgery after major weight loss in SUS is 18 years. However, for bariatric surgery, it is 16. In the literature, we found several reports on the impact of bariatric surgery on adolescents^25^ ^,^ ^26^. Elander et al. reported that the worsening of quality of life due to skin excess is even greater in adolescents when compared with adults^25^. Staalsen et al. reported that 91% of adolescents complain about their body image and 88% request reconstructive plastic surgery^26^. These findings support the discussion of increasing access to this type of procedure in Brazil and including patients in this age group.

The prevalence of body surgery among bariatric patients was 13.8%. This rate is similar to those reported in Austria and in the USA, of 13.9% and 11.3%, respectively^12^ ^,^ ^16^. Felberbauer et al. reported that these low results may be related to cultural and climatic aspects^16^. Gussenof et al. pointed out that economic aspects can influence such results, since in the US patients have access to these procedures via insurance and/or health plans, thus depending on reimbursement and the high costs of the private sector^12^.

In Brazil, several aspects may be related to the low prevalence^12^. Previous studies have shown difficulties and barriers that patients face in accessing plastic surgery in SUS, with an ineffective referral and counter-referral system and few hospitals with the capacity to offer highly complex treatments^27^ ^,^ ^28^.

Geographic disparity is another important finding in our study and reflects economic and structural differences between Brazilian regions. Previous studies on the capacity and organization of surgical services in Brazil have shown similar results. Massenburg et al. identified that the South and Southeast regions had a greater number and supply of surgical procedures and a greater trained workforce, with better distribution^8^. In addition, we observed an inequality within the states, as the procedures are concentrated in the capitals.

Since 2007, we have noticed an increase in surgeries performed until 2019. With the impact of the Sars-Cov-2 pandemic, there was a significant reduction in the number of procedures between 2020 and 2021. This is the first study to demonstrate the impact of the SARS-Cov-2 pandemic in plastic surgeries in post bariatric patients. Pagotto et al. reported a significant drop, of 74.2%, in elective plastic surgeries in a tertiary university hospital^29^. Singhal et al. carried out an online survey to assess the impact of the pandemic on bariatric surgeries, observing that 89% of respondents had a reduction greater than or equal to 50% in their services^30^.

Unlike studies that were limited to the lockdown period, we noticed a statistically significant reduction in the total number of procedures in Brazil in all months of 2020 and 2021^9^ ^-^ ^11^ ^,^ ^29^. The effect of surgical restrictions led to a decrease in supply, making access even more difficult and increasing the waiting queue for these surgeries.

The return of body contouring procedures to pre-pandemic rates is essential to maintain the multidisciplinary approach of bariatric patients by SUS, reducing psychological effects and preventing weight regain. Improving the geographic distribution, structure, and training of health professionals can be a solution to minimize the impacts of the Sars-Cov-2 pandemic on these patients, as well as to approximate the incidence of bariatric and post-bariatric surgeries.

This study is not free of limitations. The retrospective nature and use of a national database can be affected by incorrect code descriptions, input errors, and underestimation of procedure numbers. DATASUS is also not a database that allows identifying and individualizing patients, so it does not allow differentiating multiple procedures in the same individual.

## CONCLUSION

Brazil has presented a significant number of body contouring surgeries in patients with great weight loss after bariatric surgery since its inclusion in the Public Health System, although it still displays a low prevalence. Dermolipectomy was the most performed procedure. We noticed geographic disparities and the significant impact of the Sars-Cov-2 pandemic on total procedures, which may make it even more difficult for new patients to access available procedures. A better organization of the structure and availability of these surgeries is necessary to increase their prevalence.
